# Supplementary material for: PF-431396 hydrate inhibition of kinase phosphorylation during adherent-invasive Escherichia coli infection inhibits intra-macrophage replication and inflammatory cytokine release
Source: Microbiology (Reading). 2023 Jun 13;169(6):001337. doi: 10.1099/mic.0.001337 (PMC10333790; doi:10.1099/mic.0.001337)
Supplement: Supplementary material 1 [file mic-169-1337-s001.pdf]

PF-431396 hydrate inhibition of kinase phosphorylation during adherent-invasive *Escherichia coli* infection inhibits intra-macrophage replication and inflammatory cytokine release

Xiang Li[a], Michael J. Ormsby[a,#], Ghaith Fallata[a,b], Lynsey M. Meikle[a], Daniel Walker[c], Damo Xu[a,d] and Daniel M. Wall[a*]

[a]*School of Infection and Immunity, College of Medical, Veterinary and Life Sciences, Sir Graeme Davies Building, University of Glasgow, Glasgow G12 8TA, United Kingdom*

[b]*Department of Basic Science, College of Science and Health Professions, King Saud bin Abdulaziz University for Health Sciences, Jeddah 22384, Saudi Arabia*

[c]*Strathclyde Institute for Pharmacy and Biomedical Sciences, University of Strathclyde, Glasgow G4 0RE, United Kingdom*

[d]*State Key Laboratory of Respiratory Disease for Allergy at Shenzhen University, Shenzhen Key Laboratory of Allergy & Immunology, Shenzhen University School of Medicine, Shenzhen, China.*

[#]*Current address: Biological and Environmental Sciences, Faculty of Natural Sciences, University of Stirling, Stirling, FK49 4LA, United Kingdom*

**Corresponding author email: Donal.Wall@glasgow.ac.uk*

**Corresponding author address:*

Dr. Daniel M. Wall
Institute of Infection, Immunity and Inflammation
College of Medical, Veterinary and Life Sciences
Sir Graeme Davies Building
University of Glasgow
120 University Place
Glasgow G12 8TA

Supplementary Figures:

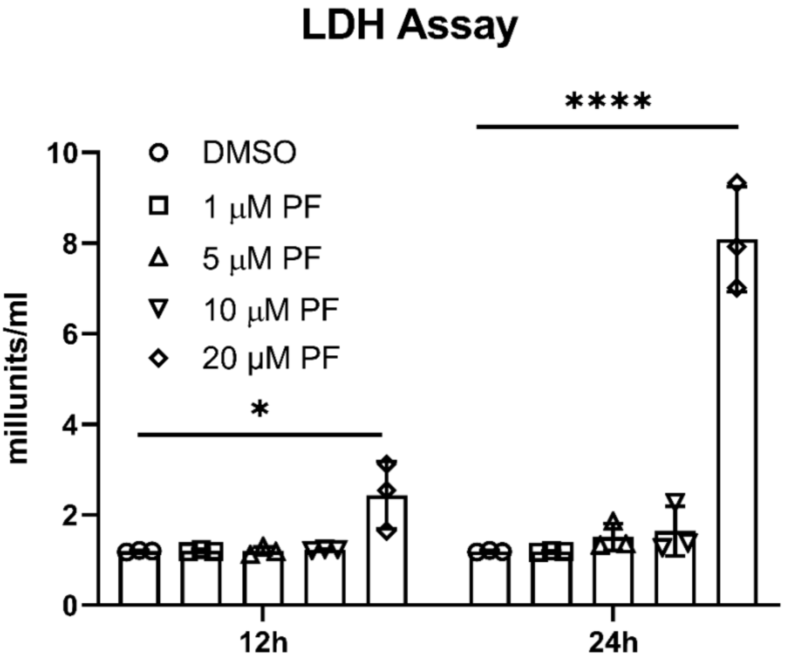

Supplementary Fig. S1. Low concentrations of Pyk2 inhibitor PF-431396 hydrate had no effect on cell toxicity. LDH activity assays were conducted on supernatants collected from RAW 264.7 cells following infection with LF82 and treatment with the Pyk2 inhibitor PF-431396 hydrate. LDH activity is reported as nmol/min/mL = milliunit/mL. Statistical analysis was conducted using a two-way ANOVA (ns, not significant, *p < 0.05, **p < 0.01, ***p < 0.001, ****p < 0.0001). Data are representative of three independent biological replicates.

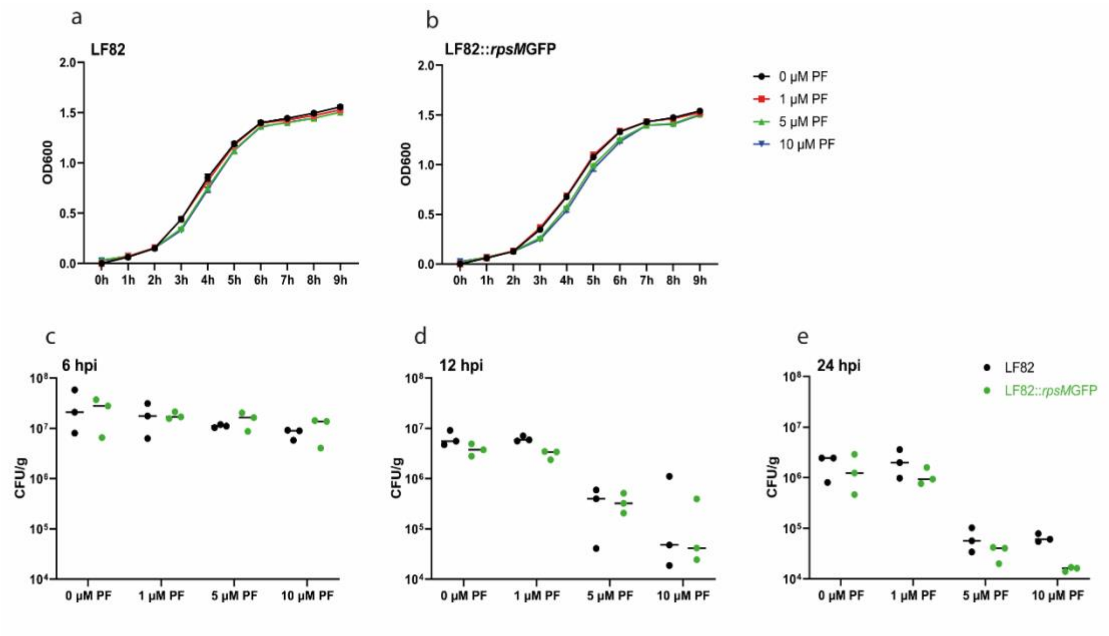

Supplementary Fig. S2: Transformation with *rpsM*GFP has no effect on growth or intracellular replication of LF82 in the presence or absence of PF-431396 hydrate. LF82 was transformed with *rpsM*GFP. Growth of LF82 (**a**) and LF82::*rpsM*GFP (**b**) was not affected by addition of increasing concentrations of PF-431396 hydrate. Intracellular replication of LF82 and LF82::*rpsM*GFP at 0 µM, 1 µM, 5 µM and 10 µM of PF-431396 hydrate was examined via viable colony counts at 6 (**c**), 12 (**d**) and 24 (**e**) hpi. Statistical analysis was conducted using a two-way ANOVA (ns, not significant, *$p < 0.05$, **$p < 0.01$, ***$p < 0.001$, ****$p < 0.0001$). Data (**c** – **e**) are representative of three independent biological replicates.

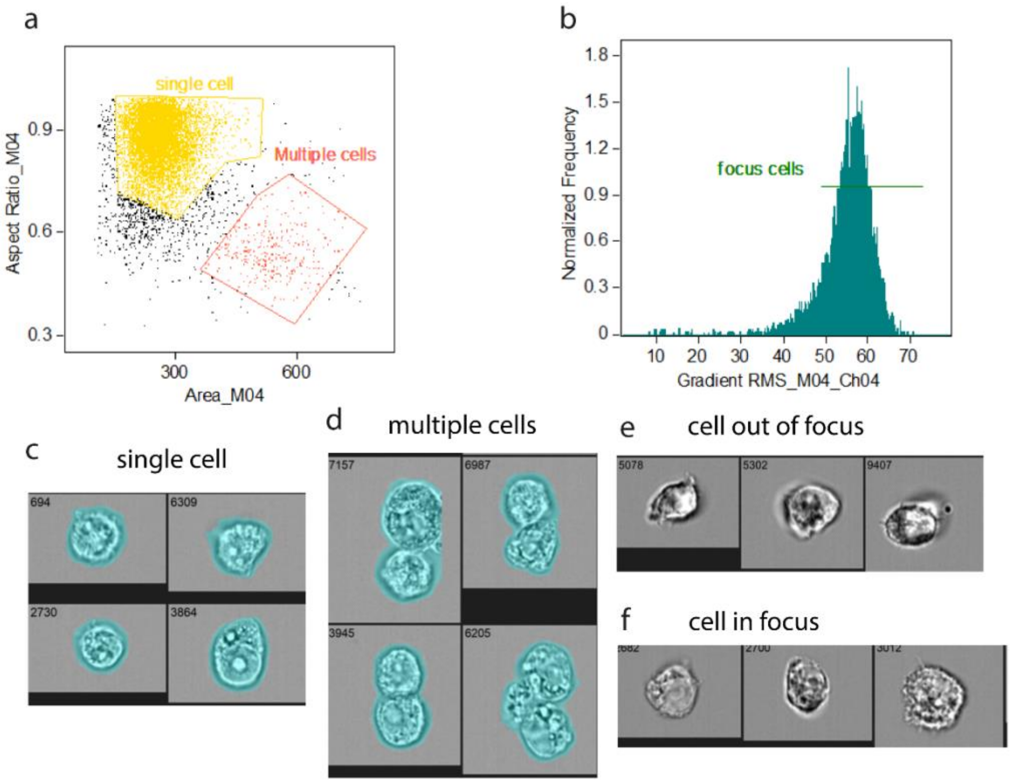

Supplementary Fig. S3: IDEAS analysis of LF82::*rpsM*GFP infected cells. RAW 264.7 were infected with LF82::*rpsM*GFP (MOI 100) for 1 h and analysed by imaging flow cytometry at 6 and 12 hpi. Single-cell population was defined by Area/Aspect ratio dot plot (**a**) and objects in best focus were gated as those events with gradient RMS values greater than 50 (**b**). Examples of cells that were included and excluded by the gating strategy; representative single cells (**c**), multiple cells (**d**), cells out of focus (**e)**; RMS value less than 50 as in [**b**]) and cells in focus (**f**; RMS value greater than 50 as in [**b**]).

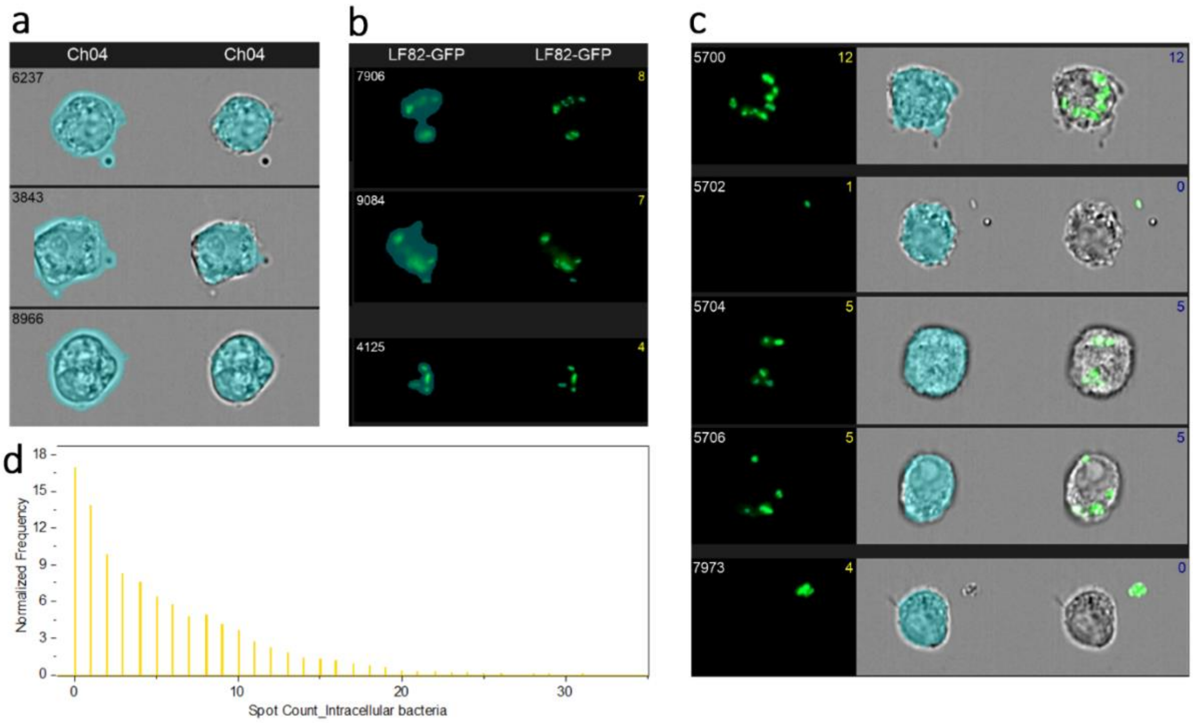

Supplementary Fig. S4: IDEAS intracellular and spot count masks. Intracellular bacterial localisation was measured by creating an intracellular mask (**a**). The spot count feature was used to quantify fluorescent spots identified using spot mask (computer code: Intensity (Peak[Spot{M02, LF82-GFP, Bright, 3.5, 3, 1}, LF82-GFP, Bright, 1]) (**b**). The spot mask in conjunction with the Erode mask was used to create an intracellular fluorescence count (**c**; computer code: Spot count-Intensity (Peak[Spot{M02, LF82-GFP, Bright, 3.5, 3, 1}, LF82-GFP, Bright, 1]) And AdaptiveErode (M04, Bright Field, 87, LF82-GFP, 80-4095). Quantitative distribution of GFP positive, single focused cells (RMS > 50) inside LF82-infected cells (**d**).
